# VACTERL association in a fetus with multiple congenital malformations – Case report

**DOI:** 10.25122/jml-2021-0346

**Published:** 2021

**Authors:** Paul Costin Pariza, Irina Stavarache, Vasile Adrian Dumitru, Octaviana Munteanu, Tiberiu Augustin Georgescu, Valentin Varlas, Consuela-Mădălina Gheorghe, Roxana Elena Bohîlțea

**Affiliations:** 1. Department of Obstetrics and Gynecology, Carol Davila University of Medicine and Pharmacy Bucharest, Bucharest, Romania; 2. Department of Radiology, Fundeni Clinical Institute, Bucharest, Romania; 3. Department of Pathology, Carol Davila University of Medicine and Pharmacy, Bucharest, Romania; 4. Department of Anatomy, Carol Davila University of Medicine and Pharmacy, Bucharest, Romania; 5. Department of Obstetrics and Gynecology, Filantropia Clinical Hospital, Bucharest, Romania; 6. Department of Marketing and Medical Technology, Carol Davila University of Medicine and Pharmacy, Bucharest, Romania

**Keywords:** VACTERL association, vertebral anomalies, tracheoesophageal fistula, cardiac anomalies, renal anomalies, limb anomalies

## Abstract

VACTERL represents an acronym for a broad spectrum of congenital anomalies such as vertebral anomalies, anorectal anomalies (anal atresia), cardiac anomalies, tracheoesophageal fistula or atresia, renal anomalies, and limb anomalies. We present the case of a male fetus with multiple anomalies consistent with VACTERL association such as scoliosis, imperforate anus, common truncus arteriosus, tracheoesophageal fistula associated with inferior esophagus atresia, polycystic kidneys, with short right ureter, lower limb hypoplasia micrognathia, hygroma, duodenal atresia, and cloacal malformation, with an aberrant omphalomesenteric duct. The presented case highlights the crucial importance of pathologists specialized in the dissection and confirmation of fetal abnormalities as an essential part of the multidisciplinary team that establishes the management of complicated pregnancies with this type of pathology.

## Introduction

VACTERL represents an acronym for a broad spectrum of congenital anomalies such as vertebral anomalies, anorectal anomalies (anal atresia), cardiac anomalies, tracheoesophageal fistula or atresia, renal anomalies, and limb anomalies [1]. Although there are no well-accepted strict diagnostic criteria, at least three features in each category are necessary for the clinical diagnosis, without other phenotypic or genetic characteristics of an alternative diagnosis [2]. The frequency is estimated to be about 1 in 10000 to 1 in 40000 live-born infants, depending on exact criteria used in different cohorts and studies. Some studies affirmed that the condition is more frequent in males. There is no evidence for increased prevalence in different regional areas or specific ethnic populations [2, 3]. There are different risk factors in the etiology of VACTERL association, such as genetic or maternal risk factors. Maternal risk factors that play a role are maternal diabetes, assisted reproductive techniques, or chronic lower obstructive pulmonary disorders [4]. Genetic risk factors include different genes mutations or chromosomal anomalies such as deletions of 5q11.2, 6q7q35qter, distal 13q and 20q13.33, duplication of 9q and 22q11.21, supernumerary der (22) syndrome, mosaicism for supernumerary ring chromosome 12 or 18 and partial monosomy 16p13.3pter/partial trisomy 16q22qter [5]. Vertebral anomalies include hemivertebrae, dysplastic vertebrae (such as “butterfly vertebrae”, “wedge vertebrae”), vertebral fusions, and supernumerary or absent vertebrae, congenital scoliosis, caudal regression, spina bifida [6]. Anorectal anomalies consist of anal atresia (or imperforate anus), and in most cases, genitourinary anomalies are associated with most patients [6]. Cardiac anomalies include a broad spectrum ranging from minor defects to severe structural defects incompatible with life. Some defects stated in the literature are ventricular septal defects, atrial defects, Fallot tetralogy, double outlet right ventricle, atrioventricular canal defect, aorto pulmonary window, and a vascular ring [7]. Tracheoesophageal fistula can associate atresia, and ultrasonographic signs include polyhydramnios or absent gastric bubble prenatally. It can also be associated with other pulmonary anomalies [8]. Renal anomalies include vesico-urinary reflux associated with a structural anomaly, unilateral renal agenesis, dysplastic or multicystic kidneys, duplicated collecting system, hydronephrosis horseshoes kidney, renal atrophy, or hypoplasia, ectopic kidney, and isolated ureteral stenosis [9]. Limb malformations include radial anomalies, thumb hypoplasia, lower limb defects polydactyly or oligodactyly [8]. 

Solomon BD states that there are different conditions with multiple common features with VACTERL association that must be taken into consideration in differential diagnosis such as Alagille syndrome, Baller-Gerold syndrome, CHARGE syndrome, Currarino syndrome, Feingold syndrome, Fryns syndrome, Holt-Oram syndrome, Townes-Brocks syndrome, Pallister-Hall syndrome and Opitz G syndrome [1].

## Case report

**Figure 1.**
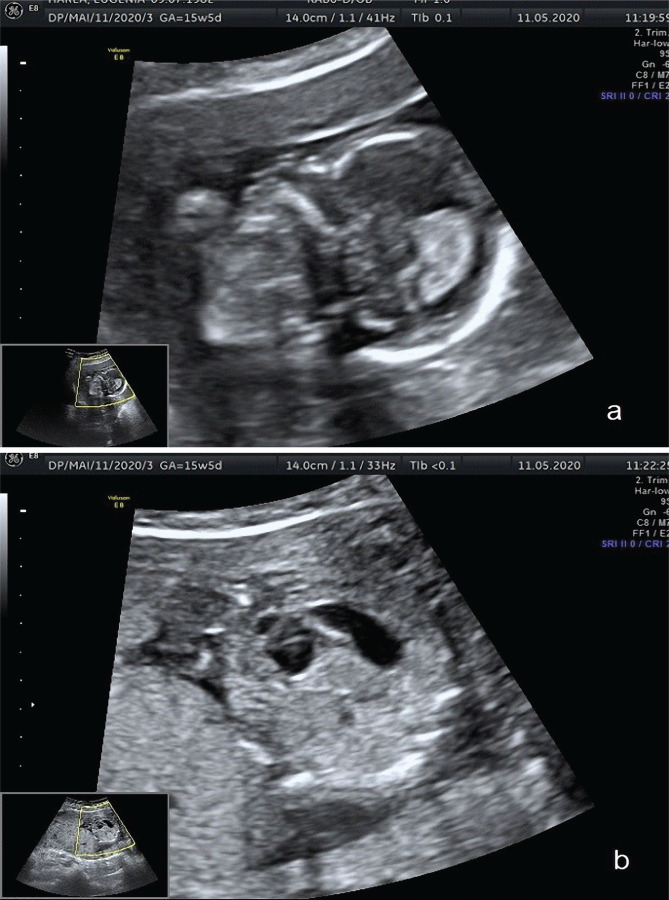

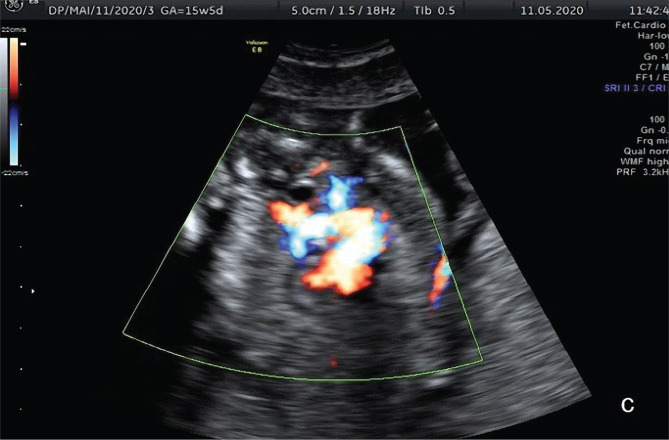
2D and color Doppler ultrasound diagnosis of micrognathia (a), double bubble sign strongly indicative for duodenal atresia (b) and common truncus arteriosus (c).

We present the case of a 38-year-old primipara pregnant woman with a medium educational level who requested an ultrasonographic evaluation at 15 weeks and 5 days gestational age. The patient’s family history included a second-degree relative who gave birth to a child with sexual ambiguity; we noted nothing abnormal from the patient’s medical history. During the 2D and color Doppler ultrasound examination, difficulty realized due to oligohydramnios, a male fetus was screened for structural anomalies, and multiple malformations were discovered, the most important being common truncus arteriosus, a single trunk that supplied both the pulmonary and systemic circulation, duodenal atresia, and micrognathia. The essential plurimalformative elements are presented in Figure 1a–c. The suspicion of VACTERL association was raised, and after appropriate counseling, the patient decided to terminate the pregnancy. No fetal karyotype test postabortum was performed. 

**Figure 2.**
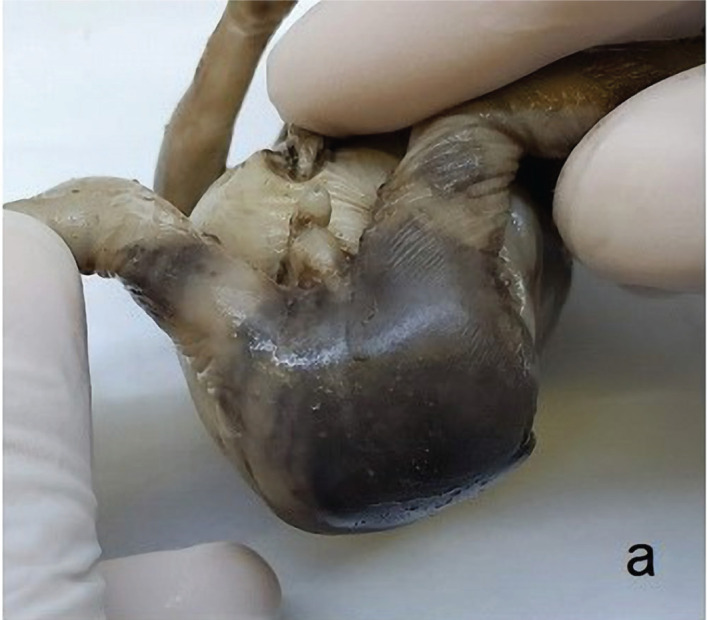

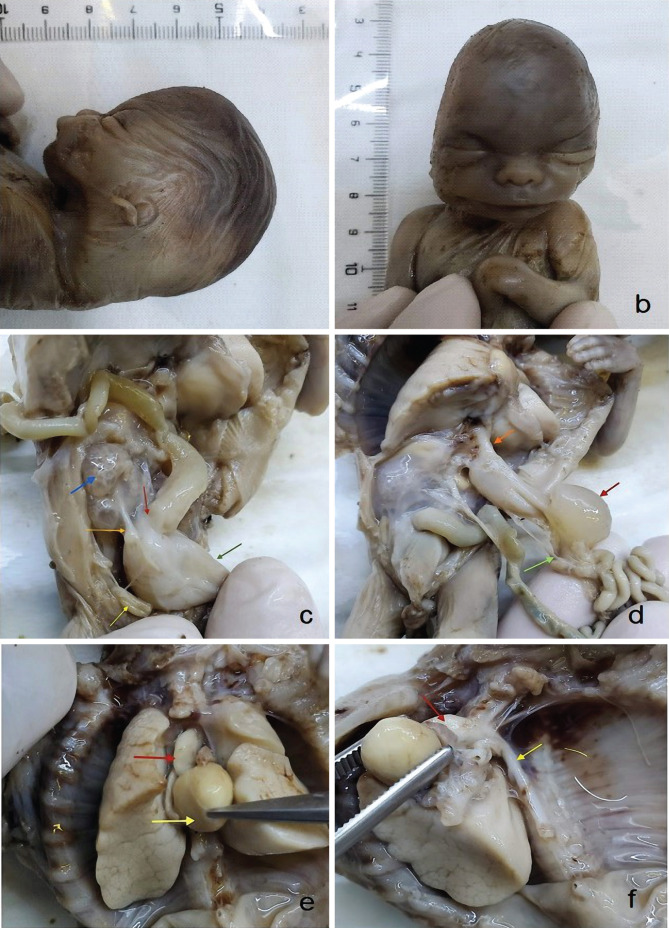

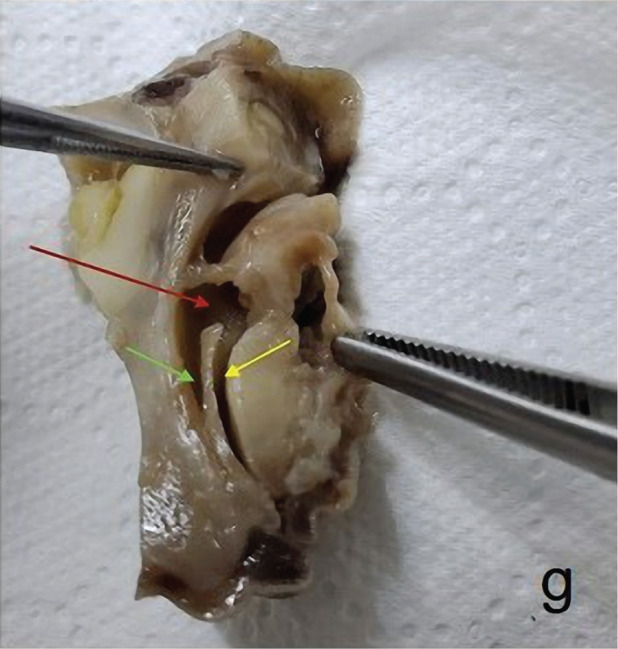
Detailed anatomopathological examination of the aborted fetus reveals masculine gender and imperforate anus (a), micrognathia and low-set ear in frontal and lateral view (b), cloacal malformation undivided by urogenital septum (green arrow) is where the sigmoid (red arrow) and short right ureter (orange arrow) open and continue inferiorly with urethra; right testis in descensus (yellow arrow) and polycystic kidney covered by renal fascia (blue arrow) (c), dilated stomach (red arrow), abdominal esophagus (orange arrow), atretic duodenum (green arrow) (d), cardiac malformation: common arterial trunk (red arrow) and heart malformed with the apex anteriorly (yellow arrow) (e), common arterial trunk (red arrow) that continues with the aortic arch and thoracic aorta (yellow arrow) (f), sagittal section of the neck, at the level of which the glottis and the trachea are observed (yellow arrow), the eso-tracheal fistula (red arrow) and the atretic esophagus towards the lower part (green arrow) are identified (g).

The typical and atypical elements of the VACTERL association are presented in Figure 2a–g, which describe in detail the anatomopathological examination of the aborted fetus. The fetus was diagnosed with multiple anomalies consistent with VACTERL association: vertebral anomalies such as scoliosis, anorectal anomalies such as imperforate anus, cardiac anomalies such as heart malrotation with anterior apex. The fetus also presented polycystic kidneys, with a short right ureter, characterized by renal anomalies and limbs malformations – lower limb hypoplasia. A tracheoesophageal fistula was described, and it was associated with inferior esophagus atresia. The oligohydramnios could be explained by the renal anomalies even in the context of the esophageal atresia, which should normally cause polyhydramnios. In addition, the anomalies also included hygroma, right descensus testis, and cloacal malformation, with an aberrant omphalomesenteric duct.

## Discussion

Imaging is mandatory for the antenatal evaluation of congenital malformations, and, in the VACTERL association, prenatal diagnosis is challenging due to many features that are not easily detectable [10]. As stated above, at least three of the main features are required for diagnosis. Vertebral anomalies are found in 60–80% of cases, anal atresia is found in almost 90% of cases, cardiac malformations can appear in 40–80% of cases, tracheoesophageal fistula is present in 50 to 80%, limb defects can appear in 50% of cases [10]. Although facial dysmorphisms are not associated with VACTERL, Carli Diana *et al.* stated in their study based on 25 patients that they found three patients with facial asymmetries and preauricular skin tags that are typically consistent with syndromes with disturbed mesodermal cell migration during early blastogenesis [8]. In our case, we also found facial dysmorphisms such as micrognathia. Carli Diana *et al.* also stated that limb involvement is a specific characteristic of the radial axis [8], while Santos Joana *et al.* mentioned that it currently encompasses other limb anomalies such as polydactyly or lower limb defects, as we found in our case [10]. Cunningha Bridget K *et al.* mentioned that the most frequent renal malformation in their study was vesicoureteral reflux in conjunction with a structural renal anomaly [9]. Ventricular septal defects represent the most common type of cardiac defect. The cardiac malformation found in our case, common truncus arteriosus, represents 1–2% of congenital heart defects [11]. Santos Joana *et al.* mentioned that cryptorchidism is not a feature frequently seen in the VACTERL association, but it has been reported previously in other studies and was also observed in our case [10].

Duodenal atresia was observed in this case. Although it is not included in the association, Fujishiro Emi *et al.* stated that duodenal atresia is seen in 6.3% of cases with the VACTERL association. The double bubble sign associating hydramnios is the pathognomonic ultrasound element [12].

Even if VACTERL is a well-known association, we still have new elements to report with the intent to complete the non-random puzzle. The presented case highlights three very important aspects regarding maternal-fetal medicine in our country: fetal anomalies are isolate reported due to the lack of functionality of the national register of fetal anomalies; first-trimester screening is opportunistic, leaving undiagnosed before second-trimester cases like the one presented, and finally, the crucial importance of pathologists specialized in the dissection and confirmation of fetal abnormalities.

## Conclusions

In conclusion, VACTERL association is a rare wide spectrum of congenital anomalies that requires a multidisciplinary team to diagnose, counsel the mother about the severity of the associated structural malformations, inform about the fetal and neonatal prognosis, as well as specialized pathologists to perform the pathology examination and correlate the ultrasonographic findings with the pathology report. A noteworthy mention is the early diagnosis of the VACTERL association in order to offer the patient the possibility of pregnancy termination safely.

## Acknowledgements

### Conflict of interest

The authors confirm that there are no conflicts of interest.

### Consent for publication

Informed consent was obtained from the participant included in this study.

### Authorship

PCP, IS, VAD, OM, TAG, VV, REB participated in the conception and design of the study. PCP, IS, VAD, OM, TAG, VV, REB participated in the acquisition of data. PCP, IS, VAD, OM, TAG, VV, CMG, REB participated in the data analysis and interpretation, drafting the manuscript and critical revision. PCP, IS, VAD, OM, TAG, VV, REB participated in the approval of the final version of the manuscript. All authors certify that they have participated sufficiently in the work to take public responsibility for the content, including participation in the concept, design, analysis, writing or revision of the manuscript.
